# Immunosuppressive properties of cytochalasin B-induced membrane vesicles of mesenchymal stem cells: comparing with extracellular vesicles derived from mesenchymal stem cells

**DOI:** 10.1038/s41598-020-67563-9

**Published:** 2020-07-01

**Authors:** M. O. Gomzikova, A. M. Aimaletdinov, O. V. Bondar, I. G. Starostina, N. V. Gorshkova, O. A. Neustroeva, S. K. Kletukhina, S. V. Kurbangaleeva, V. V. Vorobev, E. E. Garanina, J. L. Persson, J. Jeyapalan, N. P. Mongan, S. F. Khaiboullina, A. A. Rizvanov

**Affiliations:** 1 Institute of Fundamental Medicine and Biology, Kazan (Volga Region) Federal University, Kazan, Russia 420008; 2 M.M. Shemyakin–Yu.A. Ovchinnikov Institute of Bioorganic Chemistry of the Russian Academy of Sciences, Moscow, Russia 117997; 3 Department of Microbiology and Immunology, Reno School of Medicine, University of Nevada, Reno, NV USA; 4 Department of Translational Medicine, Lund University, 205 02 Malmö, Sweden; 5 Department of Molecular Biology, Umeå University, Umeå, 901 87 USA; 6 Faculty of Medicine and Health Sciences, School of Veterinary Medicine and Science, University of Nottingham, Nottingham, LE12 5RD UK; 7 Department of Pharmacology, Weill Cornell Medicine, 1300 York Ave., New York, NY 10065 USA

**Keywords:** Mesenchymal stem cells, Immunosuppression

## Abstract

Extracellular vesicles derived from mesenchymal stem cells (MSCs) represent a novel approach for regenerative and immunosuppressive therapy. Recently, cytochalasin B-induced microvesicles (CIMVs) were shown to be effective drug delivery mediators. However, little is known about their immunological properties. We propose that the immunophenotype and molecular composition of these vesicles could contribute to the therapeutic efficacy of CIMVs. To address this issue, CIMVs were generated from murine MSC (CIMVs-MSCs) and their cytokine content and surface marker expression determined. For the first time, we show that CIMVs-MSCs retain parental MSCs phenotype (Sca-1^+^, CD49e^+^, CD44^+^, CD45^−^). Also, CIMVs-MSCs contained a cytokine repertoire reflective of the parental MSCs, including IL-1β, IL-2, IL-3, IL-4, IL-5, IL-6, IL-9, IL-10, IL-12(p40), IL-13, IL-17, CCL2, CCL3, CCL4, CCL5, CCL11, G-CSF, GM-CSF and TNF-α. Next, we evaluated the immune-modulating properties of CIMVs-MSCs in vivo using standard preclinical tests. MSCs and CIMVs-MSCs reduced serum levels of anti-sheep red blood cell antibody and have limited effects on neutrophil and peritoneal macrophage activity. We compared the immunomodulatory effect of MSCs, CIMVs and EVs. We observed no immunosuppression in mice pretreated with natural EVs, whereas MSCs and CIMVs-MSCs suppressed antibody production in vivo. Additionally, we have investigated the biodistribution of CIMVs-MSCs in vivo and demonstrated that CIMVs-MSCs localized in liver, lung, brain, heart, spleen and kidneys 48 h after intravenous injection and can be detected 14 days after subcutaneous and intramuscular injection. Collectively our data demonstrates immunomodulatory efficacy of CIMVs and supports their further preclinical testing as an effective therapeutic delivery modality.

## Introduction

Mesenchymal stem cells (MSCs) are immune privileged cells characterized by low expression of MHC class I and the absence of expression of MHC class II antigens^1^. MSCs can act as immune regulators, both suppressing proliferation of activated leukocytes^2^ and inhibiting inflammation^3,4^. These immunosuppressive properties has driven intense interest in the therapeutic potential of MSCs with > 200 active clinical trials ongoing (https://clinicaltrials.gov/; accessed March 24, 2019). Indeed MSCs have been used as an immunosuppressive agent after allogeneic transplantation and in autoimmune diseases (reviewed Wang et al.^5^).

Several mechanisms were suggested to explain the immunosuppressive effect of MSCs including cell-to-cell contact, secretion of soluble factors and secretion of immune-modulatory extracellular vesicles (EVs)^6^. It is generally accepted that EVs play a fundamental role in intercellular communication, delivering biomolecules (proteins, lipids, mRNA, microRNA, etc.) to target cells^7^. EVs derived from MSCs and stromal cells emit therapeutic effects similar to that of parental MSCs^8^. It was demonstrated that MSCs derived EVs (MSCs-EVs) are immunologically active and induce expression of anti-inflammatory cytokines, decrease the level of pro-inflammatory cytokines and enhance survival of allogeneic skin graft in mice^9^. MSCs-EVs were used to treat human graft-versus-host diseases, where significant improvement of symptoms and reduction of inflammatory cytokines were revealed^10^.

**Figure 1:**
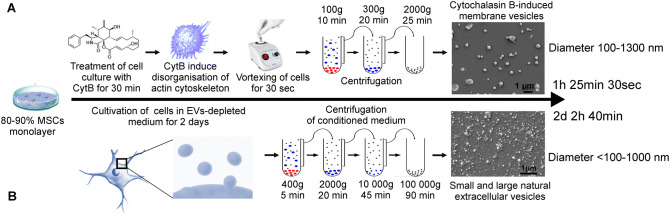
Production of cytochalasin B-induced microvesicles (**A**) and natural EVs (**B**). Schematic of isolation procedure, time required for isolation, representation of CIMVs and EVs size.

Stem cell therapies depending on the use of live cells face many challenges related to their expense, safety, difficulties in preparation, storage and shelf life^11^. In this respect, EVs provide a potential solution as they retain the biological activity, immunological properties and clinical efficacy of parental stem cells^12^. A method to increase membrane vesicle production was developed based on pharmacological disorganization of the actin cytoskeleton and vortexing^13^. Cytochalasin B-induced membrane vesicles (CIMVs) are anuclear vesicles surrounded by cytoplasmic membranes containing the cytoplasmic content of parental cells. CIMVs differ from naturally occuring EVs as there is no active mechanism of cargo sorting during CIMV generation. Cytochalasin B-induced membrane vesicles (CIMVs) have advantages over EVs as outlined previously^14^ and have been derived from numerous cell types including HEK293^13,15,16^, 3T3 fibroblast^15^, HUVECs^13^, MDCKII-MDR1^17^, SH-SY5Y^14^ and PC3 cells^18^. A comparison between isolation procedures of CIMVs and natural EVs is presented in Fig. 1.

Our previous studies also confirmed that the biological activity of CIMVs is similar to that of the parental cells^14^. Also, we have shown that CIMVs were able to stimulate capillary tube formation in vitro and vasculo-genesis in vivo by delivering vasculogenic factors^14^. Multiple studies have shown that CIMVs could serve as vehicles delivering various compounds, including anticancer drugs^19–21^. Therefore, CIMVs are emerging as novel cell-free therapeutics. However to enable further pre-clinical and future clinical testing of CMVs, it is necessary to understand the immunological properties of CIMVs-MSCs.

In this study, we therefore sought to determine the immunomodulatory properties of CIMVs-MSCs. Here, for the first time, we characterized murine CIMVs-MSCs by scanning electron microscopy, immunostaining and multiplex cytokine methods. The immune effects of CIMVs-MSCs and the MSCs were investigated by analyzing the anti-sheep red blood cells (SRBC) antibody titer, neutrophil and macrophage activity in vitro and assessing the leukocyte counts in immune organs. Additionally, organ and tissue distribution was analyzed using fluorescently labeled CIMVs-MSC in vivo.

## Materials and methods

### Animals

Adult mice (*Mus musculus*, C57Bl/6) (Pushchino, Russia) were used for experiments. All experiments were carried out in compliance with the procedure protocols approved by Kazan Federal University local ethics committee (protocol #5, date 27.05.2014) according to the rules adopted by Kazan Federal University and Russian Federation Laws. For immune response analysis, mice received intravenous injection of 7.5 × 10^4^ MSCs, 15 µg of CIMVs-MSCs or MSCs-derived EVs. Each experimental group contained five animals. All experiments were repeated three times. Mice were euthanized in compliance with the procedure protocols approved by Kazan Federal University local ethics committee (protocol #5, date 27.05.2014).

### MSCs isolation and characterization

MSCs were isolated from subcutaneous adipose tissue of the adult mice (8–9 weeks old). Adipose tissue were dissected into small pieces and digested with 0.2% collagenase II (Dia-M, Russia) in a shaker-incubator at 37 °C, 120 rpm for one hour. Suspended cells were pelleted (500 g for 5 min), washed once in PBS (PanEco, Russia) and re-suspended in DMEM (PanEco, Russia) supplemented with 10% fetal bovine serum (Gibco, UK) and 2 mM l-glutamine (PanEco, Russia). MSCs were maintained at 37 °C, 5% CO_2_ with culture medium being replaced every 3 days. MSCs from passages 3 and 4 were used for the experiments.

MSCs were differentiated into three lineages: adipogenic, chondrogenic and osteogenic and it was confirmed by detection of lipid droplets (Oil Red dye staining), glycosaminoglycans and mucins (1% alcian blue staining) and calcium deposits (5% AgNO_3_ staining), respectively. Immune phenotype was determined using monoclonal antibodies to CD90.2-PerCP (1301575; Sony, USA), CD44-APC/Cy7 (103028; BioLegend, USA), CD73-Alexa Fluor647 (127208; BioLegend, USA), CD49e-PE (1119525; Sony, USA), Sca1-APC/Cy7(108126; BioLegend, USA), CD29-PE (102208; BioLegend, USA), CD11b-PE/Cy7 (101216; BioLegend, USA), CD10-PE (312203; BioLegend, USA), CD45-PE/Cy7 (103114; BioLegend, USA). Expression of CD markers was analyzed by flow cytometry (BD FACS Aria III (BD Bioscience, USA)).

### CIMVs production

CIMVs were prepared as described previously^14^. Briefly, MSCs (passage 3–4) were washed twice with PBS, and maintained in DMEM supplemented with 10 µg/ml of Cytochalasin B (Sigma-Aldrich, USA) for 30 min (37 °C, 5% CO_2_). A cell suspension was vortexed vigorously for 30 s and pelleted (100 g for 10 min). The supernatant was collected and subjected to two subsequent centrifugation steps (300×*g* for 20 min and 2000×*g* for 25 min). The CIMVs-MSC pellet was washed once (PBS, 2000×*g* for 25 min) before use.

### EVs isolation

Murine MSCs were seeded 1 × 10^6^ cells per 10 cm^2^ and incubated overnight. Following the overnight incubation, cells were washed in phosphate-buffered saline (PBS) and fresh media containing EV-depleted FBS (Gibco, UK) was applied. EV-depleted FBS was obtained by centrifugation at 120,000×*g* for 18 h at 4 °C. Cells were incubated for 48 h under standard conditions. After 48 h, the media was collected. The conditioned medium was centrifuged at 400×*g* for 5 min at 4 °C. The resulting supernatant was sequentially centrifuged at 2,000×*g* for 20 min at 4 °C and 10,000×*g* for 45 min. Then the supernatant was transferred to ultracentrifuge tube and centrifuged at 100,000×*g* for 90 min at 4 °C using SW28Ti rotor (Beckman Coulter, USA) in the BECKMAN L70 ultracentrifuge (Beckman Coulter, USA).

### Characterization of the CIMVs and EVs

#### Scanning electron microscopy

CIMVs-MSCs and MSCs-derived EVs were fixed (10% formalin for 15 min), dehydrated using graded alcohol series and dried at 37 °C. Prior to imaging, samples were coated with gold/palladium in a Quorum T150ES sputter coater (Quorum Technologies Ltd, United Kingdom). Slides were analyzed using Merlin field emission scanning electron microscope (Carl Zeiss, Germany). For size analysis, three independent batches of CIMVs were produced and used to generate at least six electron microscope images for each batch. Data collected was used to determine the CIMVs size.

#### Polymerase chain reaction (PCR)

The sequences of the primers used were: **18S rRNA**, Forward: tacctggttgattctgccagt, Reverse: attaccgcggctgct; mitochondrial *cytochrome oxidase-1 (COI),* Forward: ggtcaacaaatc ataaagatattgg, Reverse: taaacttcagggtgaccaaaaaatca (Liteh, Russia). The PCR mixture (50 µl) contained 200 ng of DNA, 1 µM of forward primer and reverse primer, 200 µM of dNTPs, 1 × PCR buffer and 1 units of Thermus thermophilus DNA polymerase (SibEnzyme, Russia). The DNA was amplified using the following thermocycling steps: 18S rRNA—94 °C for 2 min; 28 cycles of 94 °C for 30 s, 53.9 °C for 1 min; 72 °C for 1.5 min and 72 °C for 7 min; COI: 94 °C for 2 min; 28 cycles of 94 °C for 30 s, 45 °C for 1 min; 72 °C for 1.5 min and 72 °C for 7 min. PCR products were analyzed by 2% agarose gel electrophoresis and staining with ethidium bromide.

#### Flow cytometry analysis

The immune phenotype of CIMVs-MSCs and MSCs-derived EVs was characterized by immunostaining with the following monoclonal antibodies: Sca1-APC/Cy7 (BioLegend, USA), CD49e-PE (1119525; Sony, USA), CD44-APC/Cy7 (BioLegend, USA), CD45-PE/Cy7 (BioLegend, USA), CD9-APC (Biolegend, USA), CD63-FITC (Biolegend, USA). CIMVs and EVs were analyzed by flow cytometry (BD FACS Aria III. BD Bioscience, USA), the 405 nm laser was used for better distinguish CIMVs and and EVs from debris.

### Multiplex analysis

Multiplex cytokine analysis based on the xMAP Luminex technology was performed with the Bio-Plex Pro Mouse Cytokine 23-plex Assay kit which enables quantification of IL-1α, IL-1β, IL-2, IL-3, IL-4, IL-5, IL-6, IL-9, IL-10, IL-12(p40), IL-12(p70), IL-13, IL-17, CCL11, G-CSF, GM-CSF, IFN-γ, KC, CCL2, CCL3, CCL4, CCL5 and TNF-α) (BioRad, USA), in accordance with the manufacturer's instructions. Briefly, samples were incubated with fluorescent beads for 1 h, washed and incubated with phycoerythrin-streptavidin for 10 min (PanEco, Russia). The analysis was done using a Bio-Plex^®^ 200 Systems analyzer (BioRad, USA).The CIMVs-MSC and MSCs lysates were prepared using IP buffer (50 mM Tris–Cl, 150 mM NaCl, 1% Nonidet-P40) and used for multiplex analysis. Equal protein amounts were used the analysis.

### Immunization and antibody titer analysis

To evaluate the immunological properties of MSCs and MSCs derived CIMVs mice were pretreated with MSCs, CIMVs-MSCs or MSCs-derived EVs by i.v. injection. The amount of MSCs was chosen based on literature about the amount of MSCs for the treatment of internal organ injury^22^. CIMVs and EVs were used at a concentration equivalent to 7.5 × 10^4^ MSCs based on total protein concentration. Mice were pretreated with MSCs (7.5 × 10^4^/mouse), CIMVs-MSCs (15 µg/mouse) or EVs (15 µg/mouse) 1 h before the intraperitoneal (i.p.) immunization with Sheep Red Blood Cells (2 × 10^7^; SRBC) (Biodiagnostica, Russia). Controls were injected with sterile saline (0.9%). Blood samples were collected four days post-immunization and used for serum separation. Serum anti-SRBC antibody titer was analyzed using agglutination assays. Briefly, serial serum dilutions were incubated with equal volume of sheep erythrocytes (1%; PBS) for 2 h 37 °C. Erythrocyte agglutination was visually detected by appearance of SRBC clumps. The agglutination titer was calculated as follows: Titer = log2^n^; n = the last dilution when agglutination was detected.

### Chemiluminescence test

Functional activity of neutrophils was analyzed by detection of reactive oxygen species (ROS). Neutrophils were isolated from heparinized blood by density gradient separation (histopaque 1.077 g/cm^3^ and 1.119 g/cm^3^, Sigma, USA) 30 min at 1,500 rpm. The intermediate phase was collected and washed twice (PBS; PanEco, Russia). Neutrophils (5 × 10^5^ cells/ml) were seeded onto wells containing 0.65 mM luminol in DMEM (PanEco, Russia). The level of spontaneous chemiluminescence (I_sp_—luminescence spontaneous) was measured for 1 min (37 °C under constant shaking) by TecanNanoquant Infinite 200 Pro (Tecan, Switzerland). The level of opsonized zymosan (10 mg/ml) induced chemiluminescence was detected during next 90 min.

### Phagocytic activity of peritoneal macrophages

Macrophages were collected from intraperitoneal lavage using 5 ml of DMEM (PanEco, Russia) medium supplemented with 20% fetal bovine serum (Gibco, UK) and 2 mM l-glutamine (PanEco, Russia). Medium was aspirated and macrophages (0.3 × 10^6^ cells/ml) were incubated for 4 h in a 24-well plate (37 °C, 5% CO_2_). Culture medium was removed, neutral red solution (0.075% in PBS) (Sigma, USA) was added and cells were incubated for 1 h (37 °C, 5% CO_2_). At the end of the incubation, culture medium was removed and cells were lysed in a mix of ethanol:0.01% acetic acid (1:1). Cells lysate was used to determine the optical density at 540 nm by TecanNanoquant Infinite 200 Pro (Tecan, Switzerland). Phagocytic index (PI) was calculated as:

$$ {\text{PI }} = \left( {\text{Optical density of the lysate}} \right)/({\text{number of cells}}). $$

### Leukocyte counts

Spleen, thymus and bone marrow were collected and used to determine the mass of the organ. Single-cell suspension was prepared using each organ by gentle rubbing the tissue against the sterile nylon wire mesh (40 µm). Leukocyte number was determined as: N = (Number of cells)/(organ mass).

### Leukocyte viability

Viability of leukocytes from spleen, thymus and bone marrow was analyzed. Annexin V-PI kit (640932, Biolegend, USA) was used to stain leukocytes according to the manufacturer’s instruction. Apoptotic and necrotic cells were identified by flow cytometry (BD FACS Aria III (BD Bioscience, USA)).

### PHA-activation of PBMCs

#### PBMC isolation

All blood samples from healthy donors were collected into sodium citrate-tubes (Vacuette, USA) after a written informed consent was obtained. Human samples were collected and methods were carried out in accordance with an experimental protocol approved by the Biomedicine Ethics Expert Committee of Kazan Federal University and Republican clinical hospital (# 218, 11.15.2012) based on article 20 of the Federal Legislation on "Health Protection of Citizens of the Russian Federation" #323-FL, 21.11.2011. Peripheral blood mononuclear cells (PBMCs) were isolated from anticoagulated blood via Ficoll (PanEco, Russia) gradient centrifugation.

#### CFDA SE staining of PBMC

Cell permeant dye carboxyfluorescein diacetate succinimidyl ester (CFDA-SE) (65-0850-84, eBioscience, USA) was used to trace proliferation of cells. Staining was done in accordance with the manufacturer's instructions. Briefly, PBMC (1 × 10^6^ cells/ml) were incubated 15 min with 10 µM of CFDA SE (65-0850-84, eBioscience, USA) and washed with RPMI supplemented with 10% FBS, 2 mM l-glutamine.

#### PHA-activation

Phytohemagglutinin (PHA) (PanEco, Russia) was used to induce activation and proliferation of lymphocytes in vitro. PBMCs were seeded in a 96 well-plate 1.5 × 10^5^ cells per well and preincubated with CIMVs-MSCs (10 μg per well) for 24 h in complete medium (RPMI with 10% FBS, 2 mM l-glutamine). After the incubation 10 μg/ml PHA was added to the PBMC culture. Three days later PBMCs were washed once with PBS and stained with monoclonal antibodies.

### CIMVs-MSCs biodistribution

CIMVs-MSCs were stained with lipophilic dye 1,1-Dioctadecyl-3,3,3,3 tetramethylindodicarbocyanine (DiD) (Life Technologies, USA) according to the manufacturer’s instruction to visualize CIMVs in vivo in the biodistribution experiment. CIMV suspension (300 μg/ml) was incubated with 5 µM of DiD dye for 15 min (37 °C, 5% CO_2_). CIMVs were washed once with DMEM (supplemented with 2 mM l-glutamine and 10% EVs-depleted FBS) and once with PBS (2000 g for 25 min) before use. EVs-depleted FBS was obtained by centrifugation at 120,000×*g* for 18 h at 4 °C. The supernatant (PBS) after the last washing step was collected and used in the biodistribution study as a control to exclude the risk of false positive signals from unbound dye. Mice were injected with DiD labeled CIMVs-MSCs intravenously (50 µg/mouse), intramuscularly (50 µg/mouse) and subcutaneously (25 µg and 50 µg per mouse) and used for fluorescent image acquisition (IVIS Imaging System (PerkinElmer, USA)). Mice received the intravenous injection were euthanized for the subsequent analysis of organs ex vivo. Mice were perfused prior to organ harvest. Fluorescent images were collected at selected time points (1 h, 48 h or 14 days). The relative fluorescence units (RFU) were measured using Living Image Software (PerkinElmer, USA).

### Statistical analysis

Statistical analysis was done using Wilcoxon signed-rank test (R-Studio) with significance level p < 0.05. Illustrations were generated with “ggplot2” package. Data on cytokine content of CIMVs-MSC and MSC were plotted as a heatmap.

## Results

### Isolation and characterization of mouse adipose-derived MSCs

**Figure 2:**
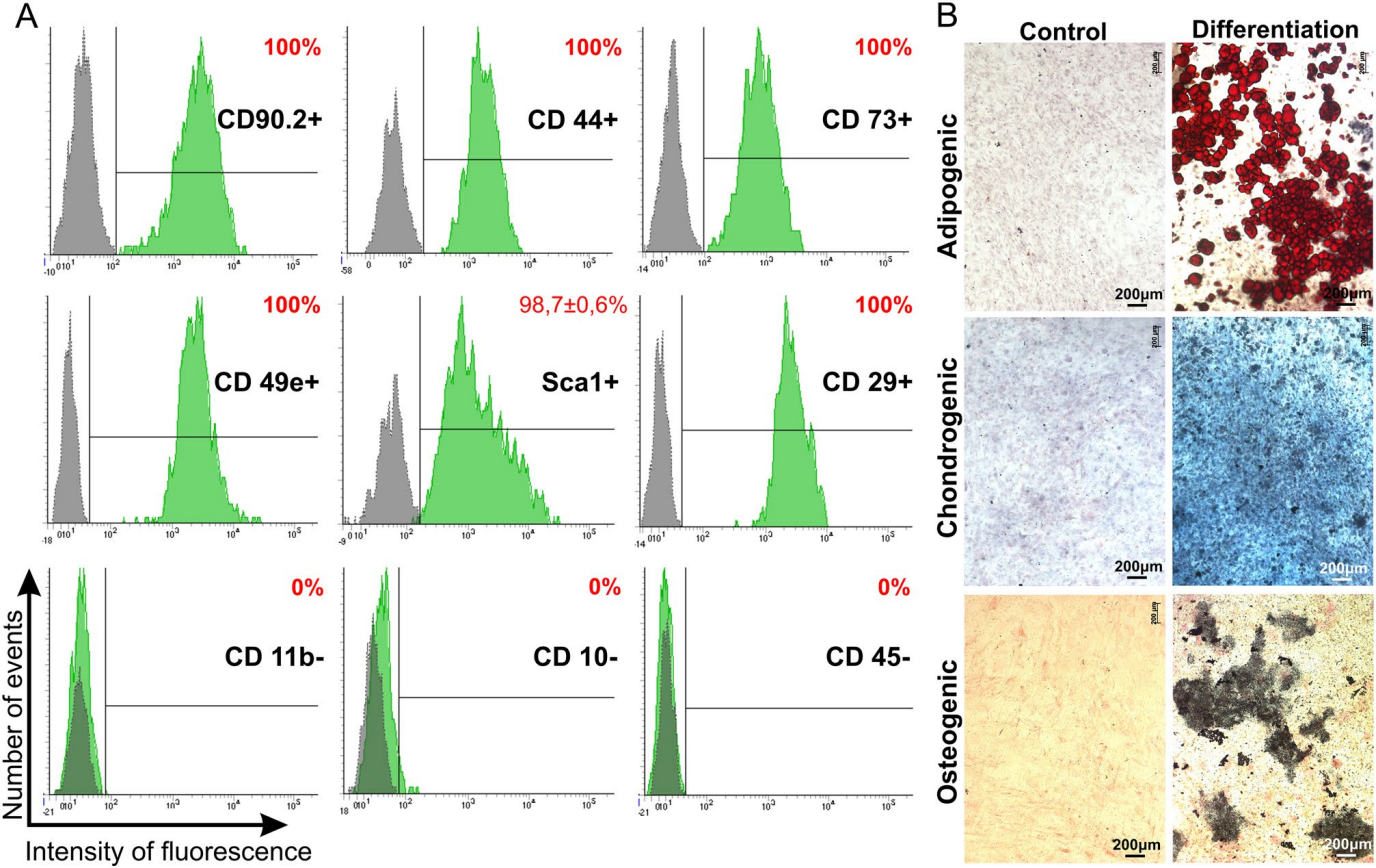
Phenotypic analysis of murine MSCs. (**A**) Flow cytometry analysis. Histograms were generated using FACSDiva7 software. Grey—negative control; Green—cells labeled with fluorescence labeled antibodies. (**B**) MSC differentiation into the adipogenic, chondrogenic and osteogenic lineages. Differentiation was revealed by following stainings: adipogenic differentiation—with Oil Red dye, chondrogenic differentiation—alcian blue, osteogenic—silver nitrate staining. Images were captured using ZEISS Axio Observer Z1 microscope.

Primary MSCs were isolated from murine subcutaneous adipose tissue. These cells had a typical murine MSCs phenotype as they expressed CD90.2, CD44, CD73, CD49e, Sca1, CD29 and lacked expression of CD11b, CD10 and CD45 (Fig. 2A)^23^. To confirm the multipotent capacity, MSCs were differentiated into the three lineages: adipogenic, chondrogenic and osteogenic (Fig. 2B).

### CIMVs-MSCs size

**Figure 3:**
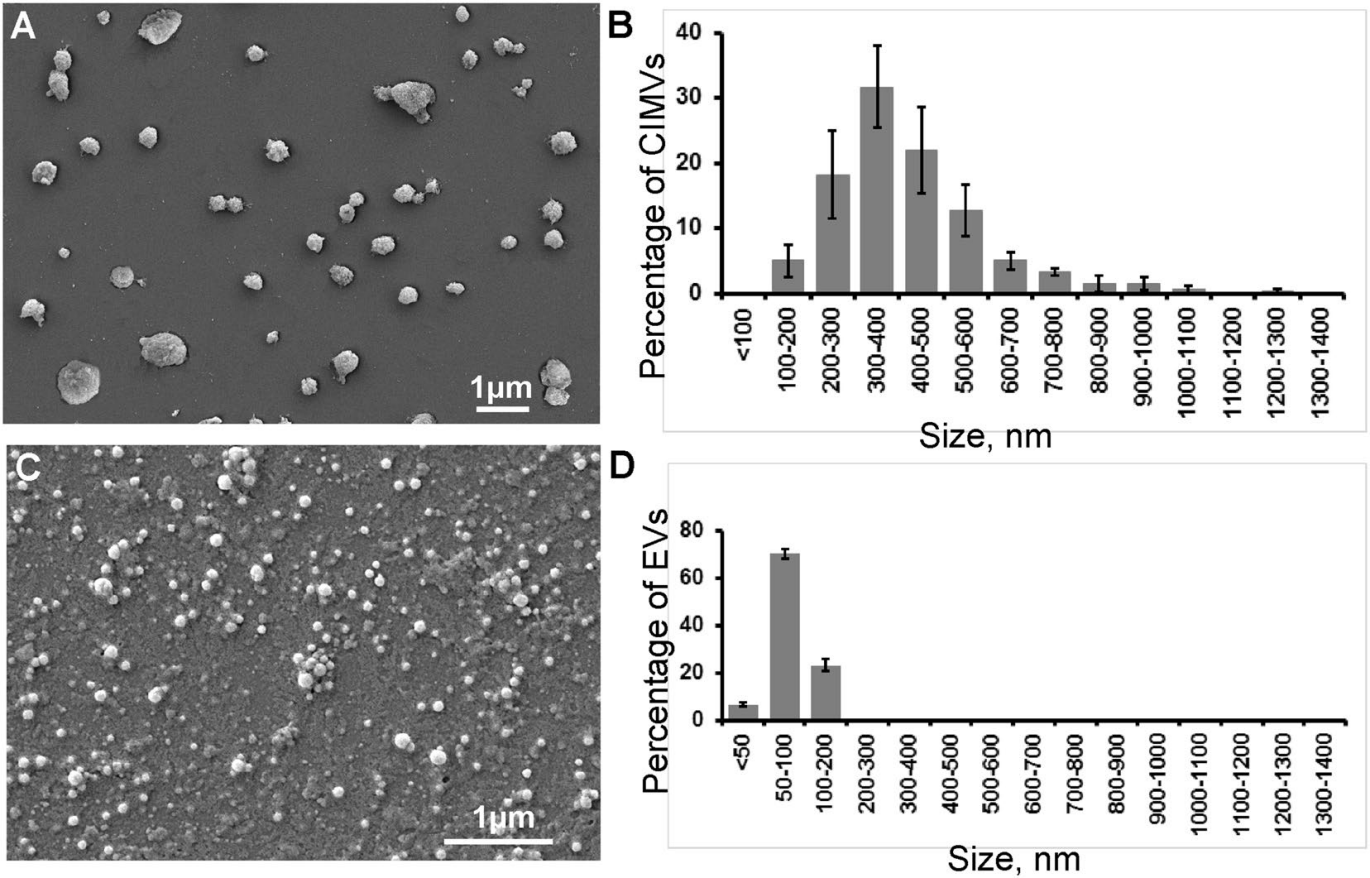
The morphology and size range of the murine CIMVs-MSCs and MSCs-derived EVs. (**A**,**C**) Scanning electron microscopy was used to characterize the murine CIMVs-MSCs and MSCs-derived EVs, respectively. Six electron microscope images were analyzed from three independent experiments to determine the size rage of the murine CIMVs-MSCs (**B**) and MSCs-derived EVs (**D**). The data represents mean ± SD.

While CIMVs have previously been derived from multiple cell lines, the ability to generate CIMVs from murine MSCs has not previously been reported. Here, we have shown that CIMVs with the size ranging from 100 to 1,300 nm could be produced using primary murine adipose MSCs (Fig. 3A,B). We have characterized EVs derived from the same murine MSCs to compare their size with CIMVs. We found that EVs derived from MSCs are from < 50 to 200 nm in size (Fig. 3C,D). Next, we characterized the EVs and CIMVs based on the presence of the CD9 and CD63 transmembrane tetraspanins proteins. We found that 76.8 ± 2.1% MSCs express CD9 and 15.9 ± 1.2% express CD63, whereas 46.9 ± 5% of CIMVs-MSCs are CD9 positive, 6.8 ± 1.1% of CIMVs-MSCs are CD63 positive, and 30.8 ± 4.8% of EVs are CD9 positive and 27.9 ± 4.1% of EVs are CD63 positive (Supplementary Fig. S1). In addition, characterization of nucleic acid content in CIMVs was performed using PCR (Supplementary Fig. S2). We found that MSCs derived CIMVs contain *COI* DNA indicating the presence of mitochrondia and their components, and but do not contain *18S* rRNA DNA (marker of nuclear DNA) (Supplementary Fig. S2).

### Molecular characteristics of murine CIMVs-MSCs

**Figure 4:**
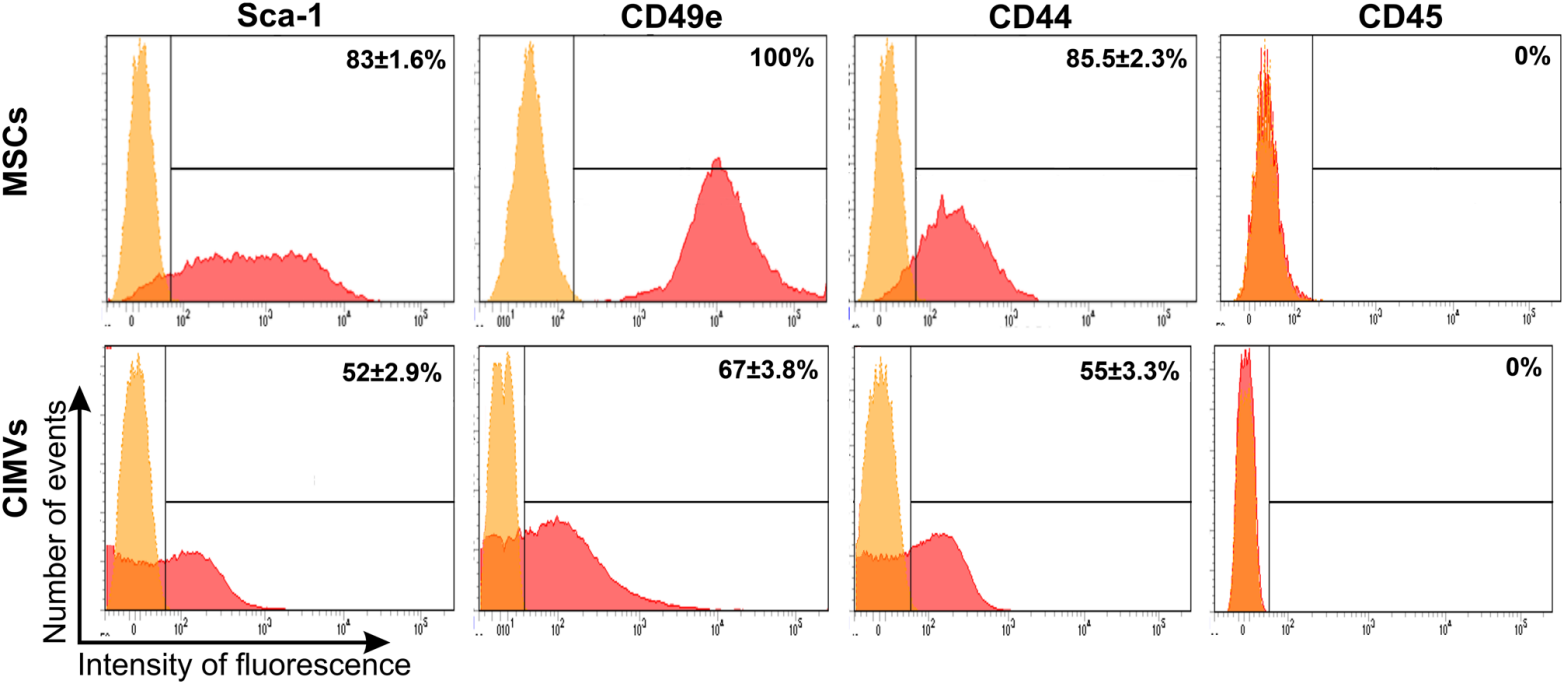
Surface receptors expressed on the murine MSCs and CIMVs-MSCs. MSCs and CIMVs-MSCs were stained with anti-Sca-1, anti-CD49e, anti-CD44 and anti-CD45 monoclonal antibodies and analyzed using flow cytometer BD FACS Aria III. Histograms were generated using FACSDiva7 software. Orange—negative control; Red—MSCs or CIMVs-MSCs labeled with antibodies.

Cell surface receptors play important role in intracellular communication and induction of receptor-mediated signaling, which is essential for CIMVs-MSCs function. Therefore, we sought to determine the repertoire of CIMVs-MSCs surface receptors. Surface markers Sca-1, CD49e and CD44 are characteristic for murine stem cells^24^. We also found that CIMVs-MSCs and parental MSCs have similar surface receptor expression (Fig. 4). Flow cytometry analyses revealed that the murine CIMVs-MSCs express following the receptors: Sca-1 (52 ± 2.9% positive), CD49e (67 ± 3.8% positive) and CD44 (55 ± 3.3% positive) (Fig. 4). Murine CIMVs-MSCs retain surface receptors similar to that of the parental MSCs.

**Figure 5:**
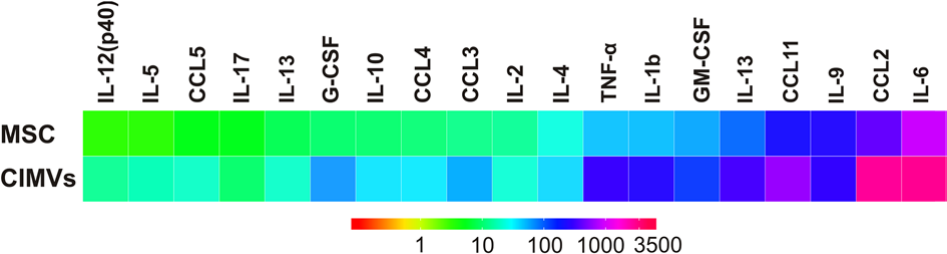
The heat map analysis of the cytokine levels in MSCs and CIMVs-MSCs.

A multiplex approach was used to analyze the cytokine content of CIMVs-MSCs. We found that the cytokine repertoire within the CIMVs-MSCs were similar to that of the parental MSCs, though cytokine levels were enriched in CIMVs-MSC as compared to the parental MCS (Table 1). Levels of IL-1α, IL-12(p70), IFN-γ, KC were below the detection range in MSCs and CIMVs-MSCs. Heat map analysis revealed that the mean expression of IL-1β, IL-2, IL-3, IL-4, IL-5, IL-6, IL-9, IL-10, IL-12(p40), IL-13, IL-17, CCL11, G-CSF, GM-CSF, CCL2, CCL3, CCL4, CCL5 and TNF-α in CIMVs-MSCs was 1.3–7.4 folds higher as compared to that in MSCs (Fig. 5). The most striking differences were observed in levels of IL-1β, IL-5, IL-6, IL-12(p40), G-CSF, CCL2, CCL3, CCL5 and TNF-α, which were 3–6.4 times higher in CIMVs-MSCs as compared to MSCs (Table 1). Therefore, we suggest that CIMVs-MSCs generation was associated with cytokine enrichment.

**Table 1:** Cytokine analysis of MSCs and CIMVs-MSCs content.

Cytokine	MSCs (pg/ml)	CIMVs-MSCs (pg/ml)	Enrichment	P value
IL-1β	60.21 ± 18.30	244.82 ± 8.13	4.1	< 0.003
IL-2	12.97 ± 1.51	25.97 ± 5.20	2.0	< 0.04
IL-3	8.30 ± 0.11	22.02 ± 2.34	2.7	< 0.007
IL-4	21.97 ± 3.19	40.10 ± 1.80	1.8	< 0.01
IL-5	4.34 ± 1.16	18.79 ± 1.57	4.3	< 0.005
IL-6	804.62 ± 127.98	3,084.32 ± 130.92	3.8	0.002
IL-9	202.76 ± 42.74	269.81 ± 18.80	1.3	< 0.09
IL-10	10.26 ± 0.26	33.10 ± 3.54	3.2	0.006
IL-12(p40)	3.20 ± 0.27	13.53 ± 0.24	4.2	< 0.0003
IL-13	100.92 ± 17.87	326.62 ± 8.09	3.2	< 0.002
IL-17	5.37 ± 0.13	12.46 ± 3.26	2.3	< 0.046
CCL11	226.23 ± 34.20	562.91 ± 57.53	2.5	< 0.01
G-CSF	10.51 ± 0.48	60.43 ± 1.78	5.7	< 0.0003
GM-CSF	52.39 ± 5.28	130.50 ± 2.19	2.5	< 0.001
CCL2	375.17 ± 37.56	2,764.96 ± 117.58	7.4	< 0.0007
CCL3	10.79 ± 2.79	50.17 ± 5.43	4.6	< 0.006
CCL4	11.12 ± 0.04	33.06 ± 2.11	3.0	< 0.0023
CCL5	5 ± 0.49	18.86 ± 1.57	3.8	< 0.0035
TNF-α	54.98 ± 12.95	314.19 ± 23.26	5.7	< 0.003

### Immunological properties of murine MSCs and CIMVs-MSCs

**Figure 6:**
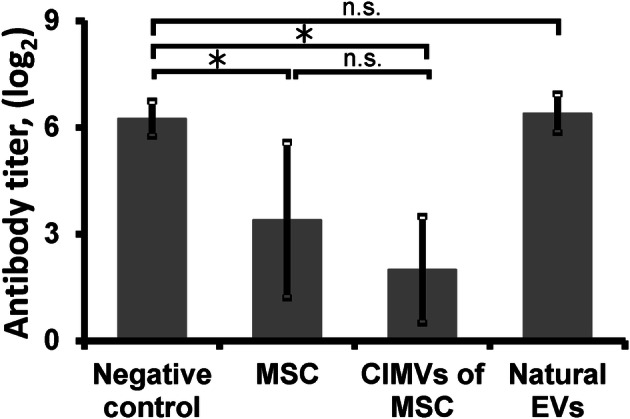
Anti-SRBC antibody titer (log2) in the serum of mice pretreated with allogenic MSCs (7.5 × 10^4^/mouse) or CIMVs-MSCs (15 µg/mouse). MSCs or CIMVs-MSCs were injected i.v. 1 h before the i. p. immunization with 2 × 10^7^ SRBC. Serum samples were collected 4 days later. Anti-SRBC antibody titer was determined using agglutination assay. The data represents mean ± SD. *p < 0.05, *n.s.* not significant.

The immunomodulatory properties of the allogenic MSCs, CIMVs-MSCs and EVs were analyzed using the SRBC murine immunization model. MSCs (7.5 × 10^4^/mouse), CIMVs-MSCs (15 µg/mouse) or EVs derived from MSCs (15 µg/mouse) were injected intravenously prior to SRBC immunization. The effect of MSCs, CIMVs-MSCs and EVs on humoral immunity was analyzed by evaluation of anti-SRBC antibodies titer using agglutination tests. We found that the antibody titer in control, saline-treated mice was 6.25 ± 0.5 (Fig. 6). The antibody titer in mice pretreated with 7.5 × 10^4^ allogenic MSCs was 3.4 ± 2.2 (p = 0.04), while pretreatment with 15 μg of CIMVs-MSCs reduced the antibody titer to 2.0 ± 1.5 (p = 0.0002) (Fig. 6), the antibody titer in mice pretreated with natural EVs was 6.4 ± 0.55 (Fig. 6). We observed that MSCs and CIMVs-MSCs suppressed the antibody production, whereas no difference between control and mice pretreated with natural EVs was observed (Fig. 6).

Next, we sought to determine the effect of the allogenic MSCs or CIMVs-MSCs on the neutrophil activity. Neutrophils were isolated from mice following intravenous injection of allogenic MSCs (7.5 × 10^4^/mouse) or CIMVs-MSC (15 µg/mouse). Neutrophils were activated with opsonized zymosan (10 mg/ml) in vitro*.* The release of the reactive oxygen species (ROS) was detected by chemiluminescence and used to determine neutrophil activation (Supplementary Fig. S3A). There was no difference between leukocyte activation in mice treated with MSCs or CIMVs-MSCs as compared to control mice, indicating that neither MSCs or CIMVs-MSCs affected neutrophil activity. The level of spontaneous chemiluminescence and the maximum value of stimulated chemiluminescence are summarized in Supplementary Fig. S3B.

Finally the effect of MSCs and CIMVs-MSCs on macrophage activity was quantified. Macrophages were isolated by peritoneal lavage from mice pretreated with MSCs, CIMVs-MSCs or sterile saline (negative control). Macrophage phagocytic activity was determined by neutral-red dye uptake. We found that the phagocytic index (PI) of macrophages from the negative control was 0.52 ± 0.14, while it was lower in cells from mice pretreated with MSCs (0.39 ± 0.07; p = 0.23) or CIMVs MSC (0.34 ± 0.13; p = 0.4) (Supplementary Fig. S4) this was not statistically significant.

The effect of MSCs and CIMVs-MSCs on hemato-lymphoid tissues was determined by leukocyte count in the spleen, thymus and bone marrow. We found that the total leukocyte count in the spleen of mice received MSCs or CIMVs-MSCs was 1,198.56 ± 157.30 × 10^6^ cells (p = 0.11) and 1,079.78 ± 137.64 × 10^6^ cells (p = 0.13), respectively, which was lower than that in control animals (1526.19 ± 315.00 × 10^6^ cells) (Supplementary Fig. S5), though this did not reach significance. The total leukocyte count in the thymus of MSCs mice received MSCs or CIMVs-MSCs was 733.16 ± 268.82 × 10^6^ cells (p = 0.11) and 773.69 ± 255.50 × 10^6^ cells (p = 0.4), respectively. Similar to that of spleenocyte count, thymocyte numbers were lower than that in mice that received PBS 1,133.69 ± 258.10 × 10^6^ cells, although these differences were not statistically significant (Supplementary Fig. S5). Likewise, the total leukocyte count in the bone marrow was lower in MSCs (12.10 ± 2.70 × 10^6^cells) (p = 0.88) or CIMVs-MSCs (11.07 ± 2.00 × 10^6^ cells) (p = 0.23) treated mice as compared to those that received PBS (13.98 ± 2.65 × 10^6^ cells) (Supplementary Fig. S5), although the differences were not statistically significant.

**Figure 7:**
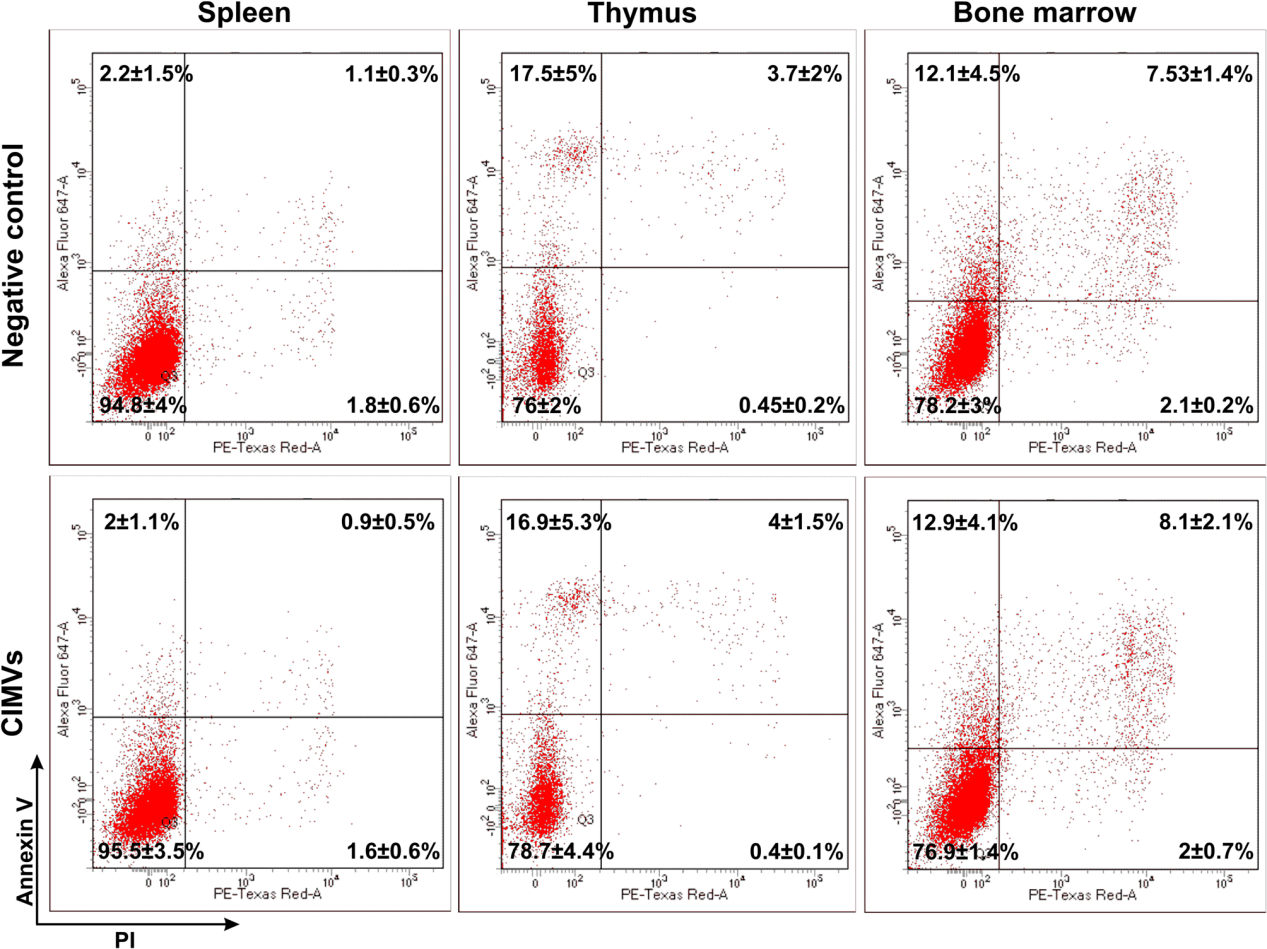
The effect of CIMVs-MSCs on viability of spleen, thymus and bone marrow leukocytes in vitro. Negative control—leukocytes incubated 24 h before the analysis; CIMVs-MSCs—leukocytes incubated with CIMVs-MSCs (15 µg) for 24 h before the analysis. Leukocytes were washed, stained with Annexin V-PI kit and analyzed by flow cytometer FACS Aria III.

The effect of CIMVs on the viability of leukocytes isolated from the spleen, thymus and bone marrow of mice was next analyzed. Leukocyte cultures were treated with CIMVs-MSCs (15 µg) for 24 h before analysis. Apoptotic and necrotic cells were detected by Annexin V-PI (640,932, Biolegend, USA) staining and followed by flow cytometry (Fig. 7). After incubation with CIMVs-MSCs, the percent of viable, apoptotic, late apoptotic and necrotic spleen leukocyte was 95.5 ± 3.5%, 2.0 ± 1.1%, 0.9 ± 0.5% and 1.6 ± 0.6%, respectively. Similar distribution of viable, apoptotic, late apoptotic and necrotic spleen leukocyte was found in the negative control cells (94.8 ± 4%, 2.2 ± 1.5%, 1.1 ± 0.3%, 1.8 ± 0.6%, respectively). Also, no differences in the viability of CIMVs-MSCs treated (76.0 ± 2.0%) and negative control (78.7 ± 4.4%) thymus leukocyte was found (Fig. 7). Similarly, the vitality of CIMVs-MSCs treated and control bone marrow leukocytes was compatible (78.2 ± 3% *vs* 76.9 ± 1.4%) (Fig. 7).

To evaluate the influence of CIMVs on activation of lymphocytes, human PBMCs have been incubated with CIMVs derived from human MSCs for 24 h and then were treated with 10 μg/ml PHA (M021, PanEco, Russia) (Supplementary Fig. S6). We observed that CIMVs-MSCs do not induce T-cells proliferation compared to control (1.6 ± 0.7% of proliferating cells vs. 1.6 ± 0.4%, respectively). Treatment of human PBMCs with PHA induced T-cells proliferation up to 87.7 ± 2%. Pretreatment of PBMCs with CIMVs led to the inhibition of PHA-activated proliferation of T-cells in 1.4 times (62.8 ± 1% of proliferating cells) (Supplementary Fig. S6).

### In vivo analysis of CIMVs-MSCs location

**Figure 8:**
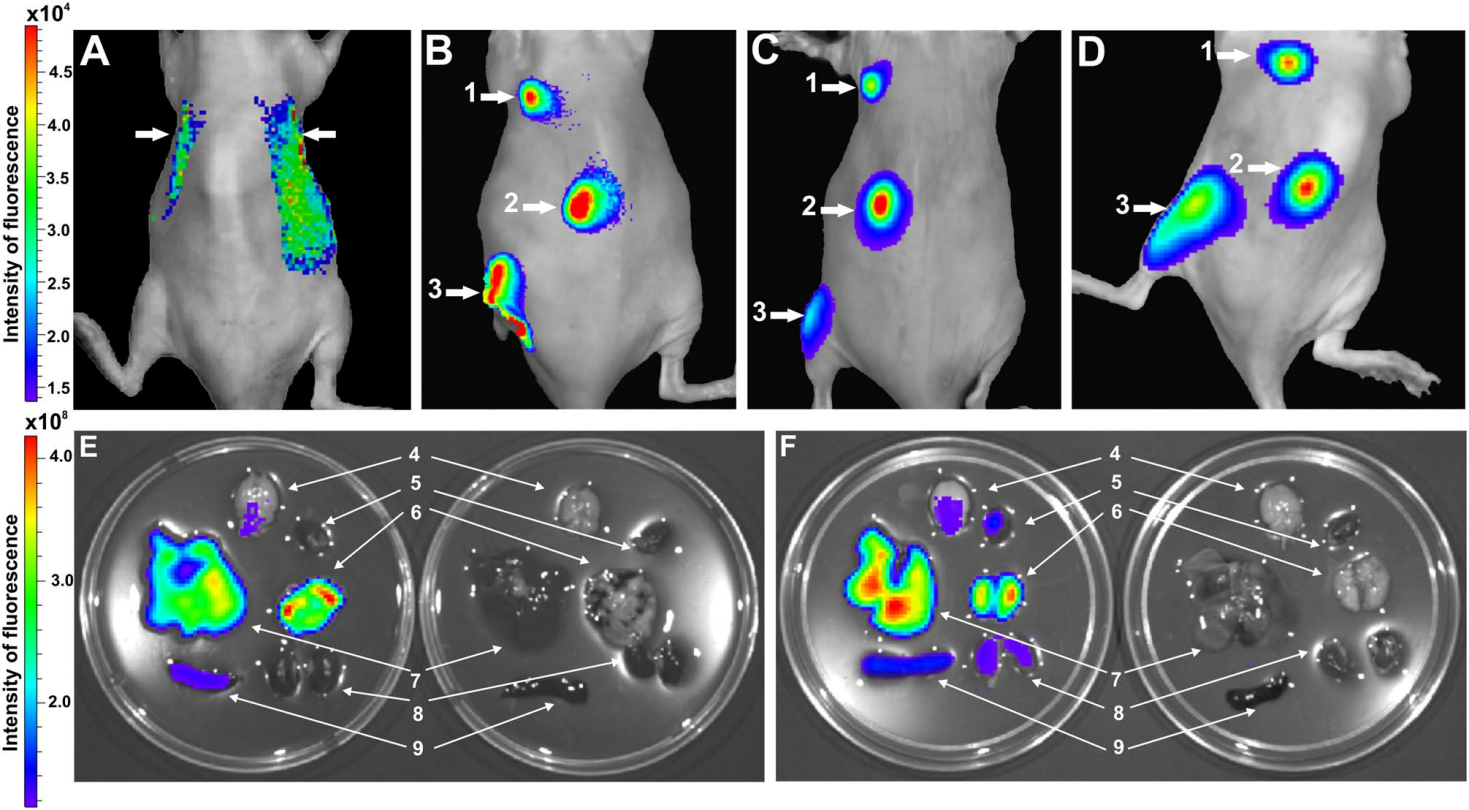
Analysis of CIMVs-MSCs distribution in vivo (**A**–**D**) and ex vivo (**E**,**F**). Biodistribution of CIMVs-MSCs after i.v. injection (**A**), s.c. injection (**B**–**D**) and i.m. injection (**B**–**D**). (**A**) 1 h after i.v. injection; (**B**) 1 h after s.c. and i.m. injection; (**C**) 48 h after s.c. and i.m. injection; (**D**) 14 days after s.c. injection; and i.m. injection; (**E**) organs were collected one hour after i.v. injection of CIMVs-MSCs (experiment; left Petri dish) or PBS (control; right Petri dish); (**F**) organs were collected 48 h after i.v. injection of CIMVs-MSCs (experiment; left Petri dish) or PBS (control; right Petri dish). 1—s.c. injection 25 µg of CIMVs-MSCs; 2—s.c injection 50 µg of CIMVs-MSCs; 3—i.m. injection 50 µg of CIMVs-MSCs, 4—brain, 5—heart, 6—lung, 7—liver, 8—kidneys, 9—spleen. Images were captured using IVIS Imaging System.

To analyze in vivo distribution, CIMVs-MSCs were stained with vital membrane dye DiD (Invitrogen, USA) and injected intravenously (50 μg), subcutaneously (25 and 50 µg) or intramuscularly (50 µg). We found that 1 h after intravenous injection of CIMVs-MSCs the fluorescence signal was localized in internal organs presumably in lung (Fig. 8A). To accurately conclude which organ the fluorescent signal originated from the organs were imaged ex vivo (Fig. 8E,F). Mice were perfused prior to organ harvest. In addition, to exclude the risk of detection of free dye per se the supernatant (PBS) after the last washing step was injected in control mice. As shown in Fig. 8E CIMVs-MSCs accumulated mainly in liver and lung, a low signal of CIMVs-MSCs was observed also in spleen and brain. At 48 h, the fluorescent signal remained in liver and lung and started to increase in brain, heart, spleen and kidneys (Fig. 8F).

It appears that the fluorescence signal intensity correlated with the amount of administered CIMVs-MSCs (Fig. 8B,D). When mice received 25 µg CIMVs-MSCs subcutaneous, the fluorescence intensity was 2.7 ± 1.2 RFU, while it was 5.5 ± 1.8 RFU (p = 0.09) when mice were injected with 50 µg of CIMVs-MSCs. Interestingly, the fluorescence signal was detectable 48 h and 14 days after subcutaneous and intramuscular injection respectively (Fig. 8C,D). These findings may be explained by CIMVs-MSCs uptake in tissue and artefacts due to the long half-life of the dye^25^.

## Discussion

Multiple methods have been used to produce membrane vesicles^26,27^. Treatment with cytochalasin B has been shown to be effective for the generation of vesicles that resemble naturally formed microvesicles in displaying physiological cytoplasmic membrane and in size^14^. EVs are isolated from extracellular medium, such as conditioned cell culture medium or body fluids^28^. Because CIMVs are produced from washed cells and their production protocol does not involve active sorting of molecules within the cells, they are distinct from natural EVs. One of the limitations of the use of CIMVs in preclinical and clinical trials is that current understanding of the immune properties of CIMVs derived from MSCs remains limited.

Recently we have shown that CIMVs can be successfully generated from primary human adipose MSCs^29^. They have similar content, immunophenotype, and angiogenic activity to those of the parental MSCs^29^. Here, for the first time, we report that murine MSCs can be used to generate CIMVs. We found that the majority of murine CIMVs-MSCs (94.75%) have a diameter of 100–700 nm, which is within the range of naturally produced microvesicles^30^. To compare the size of CIMVs and EVs we have applied the most commonly employed protocols for the EVs isolation^31^. We found that EVs are between < 50 and 200 nm in size. EV isolation protocols based on ultracentrifugation leads to the preferential enrichment of exosomes. Next, we compared the expression of CD9, CD63, which are part of the key EV criteria as agreed by the International Society of Extracellular Vesicles, on the surface of EVs and CIMVs. We found that EVs express more CD63-positive (+ 4.1-fold) and less CD9-positive (− 1.5-fold) as compared to CIMVs (Supplementary Fig. S1). It is known that CD63 is a major player in exosomes production. CD63 is often enriched in late endosomal and lysosomal compartments as well as in exosomes^32^. Whereas CD9 has an extracellular domains and is one of the markers of MSCs^33^. Therefore we confirmed the endosomal origin of EVs, whereas CIMVs production protocol involves the budding of membrane vesicles from the cell surface and as a result CIMVs enclosed by a cytoplasmic membrane.

We detected *COI* gene DNA in CIMVs which may indicate inclusion of mitochondria and derived components in the process of CIMVs production. It is known that natural EVs of BMSCs also contain mitochondria^34^. The absence of nuclei content in the CIMVs fraction indicate that CIMVs are not able to divide and might be a safer alternative than cell therapy.

The majority of CIMVs-MSCs possessed an immune phenotype similar to that of MSCs (Sca-1^+^, CD49e^+^, CD44^+^, CD45^-^). It is believed that MSCs-mediated immune-regulation is the result of the cumulative action of secreted cytokines and chemokines^35^. Therefore, the cytokine content of the murine CIMVs-MSCs was analyzed. We found that CIMVs-MSCs content was similar to that of parental MSCs including IL-1β, IL-2, IL-3, IL-4, IL-5, IL-6, IL-9, IL-10, IL-12(p40), IL-13, IL-17, CCL11, G-CSF, GM-CSF, CCL2, CCL3, CCL4, CCL and TNF-α. The level of all investigated cytokines and chemokines was higher in CIMVs-MSCs as compared to the parental MSCs.

Investigation of human MSCs secretome^36^ revealed, that G-CSF, IL-12p40, IL-17, CCL2, CCL3, CCL4 were released by MSCs in a culture medium, the level of IL-6, IL-10 and IL-13 changed non-significantly, while CCL11, GM-CSF, IL-1β, IL-2, IL-3, IL-4, IL-5, CCL5 and TNF-α were consumed by MSCs. Both human and murine MSCs contain G-CSF, IL-12p40, IL-17, CCL2, CCL3 and CCL4. However, several cytokines, IL-6, IL-10, IL-13, CCL11, GM-CSF, IL-1β, IL-2, IL-3, IL-4, IL-5, CCL5,TNF-α and IL-9, which were absent in human MSCs, were found in murine MSCs (Fig. 5, Table 1).

It was previously shown that soluble factors play a major role in the immune suppressive effects of MSCs^37^. Several cytokines found in murine MSCs could cause immune suppression. For example, IL-6 could inhibit monocyte differentiation and stimulate T cells^35^. Anti-inflammatory IL-10 could inhibit cytokine secretion, dendritic cell differentiation and antigen presentation^38^. Also, IL-4 and IL-13 could trigger macrophage differentiation into immunosuppressive M2 subset^39^. Interestingly, levels of these cytokines were increased in murine CIMVs-MSCs as compared to MSCs. The mechanism by which cytokines encapsulated in lipid vesicles influence target cells was discussed by Fitzgerald et al.^40^. It is known that cytokine receptors are located on the cell surface^41^. The authors suggested that EVs might release entrapped cytokines during the interaction with the cell surface as a result of leaky membrane formation or in the process of fusion^40^.

Similarities in the surface receptor expression and molecular content suggest that CIMVs-MSCs and MSCs could have similar immune modulatory activity. Therefore we analyzed the effect of CIMVs-MSCs and MSCs on humoral immune response in mice. Our data revealed that CIMVs-MSCs and MSCs suppress antibody production (Fig. 6). The antibody titers to SRBC in mice pretreated with allogenic MSCs or CIMVs-MSCs were lower than the antibody titer in the serum of control mice. The immune inhibitory effect could be explained by the high level of anti-inflammatory cytokine content of CIMVs-MSCs.

Previously Budoni et al.^42^ demonstrated the inhibitory effects of EVs on B-cell proliferation and antibody production. Therefore we compared the immunomodulatory effect of MSCs, CIMVs and EVs. We observed no immunosuppression in mice pretreated with natural EVs, whereas MSCs and CIMVs-MSCs suppressed antibody production (Fig. 6). Immunosuppressive activity of EVs-MSCs has been actively discussed and few authors demonstrated lower/or absence of inhibitory effect compared to parental MSCs. Our findings support the results from Conforti and colleagues showing that MSCs were significantly more capable to inhibit T-cell proliferation and antibody secretion in vitro compared to EVs^43^. Gouveia de Andrade and colleagues observed that EVs derived from bone marrow (BM-MSCs) and adipose tissue (AT-MSCs) failed to suppress lymphocyte proliferation^44^. Trapani and colleagues observed that the immunosuppression effects of EVs was less dramatic compared to the MSCs and was proportional to their uptake by immune cells population^45^. It is believed that observed contrasting action of EVs-MSCs is due to method of isolation, purity and medium size of isolated EVs^44^. In this context CIMVs might be more relevant substitute for MSC administration combining advantages of safety and ease of production with retaining parental MSCs immunomodulatory activity. In addition^36^, we observed a modest macrophage suppressive effect of CIMVs-MSCs. These data suggests that CIMVs-MSCs have inhibitory effect similar to MSCs.

It has been shown that the hemato-lymphoid system is highly sensitive to inhibitory stimuli^46^. Therefore, we analyzed the effect of CIMVs-MSCs and MSCs on the leukocyte count in major lymphoid organs such as spleen, thymus and bone marrow (Supplementary Fig. S5). Leukocyte counts in all lymphoid organs were decreased as compared to control. Although these differences did not reach statistical significance, further studies are required to determine the effect of prolonged CIMV-MSC treatment on leukocyte function and number. The changes in the leukocyte counts were not caused by the cell death, since the viability of leukocytes isolated from the spleen, thymus and bone marrow was not affected by incubation with CIMVs-MSCs (Fig. 7). This data corroborates our previous publication where human CIMVs-MSCs also did not affect the cell viability^14^. Therefore, decreased antibody production in mice receiving MSCs and CIMVs-MSCs is more likely attributable to an immunomodulatory mechanism as opposed to decreased leukocyte count. MSCs and CIMVs linked immunosuppression in mice without effecting leukocyte viability suggests that the hemato-lymphoid cells suppression was due to the inhibition of proliferation and/or differentiation.

Recently Khare et al. demonstrated the inhibitory effect of MSCs derived natural EVs on the proliferation of activated PBMCs and isolated T and B cells^47^. Therefore, we performed T-cell suppression assay in vitro to evaluate the influence of CIMVs on T-cells. We observed the inhibitory effect of allogeneic CIMVs-MSCs on PHA-activated proliferation of T-cells in 1.4 times.

For the first time we have demonstrated the bio-distribution of murine CIMVs-MSCs in vivo following intravenous, subcutaneous and intramuscular injection. We have found that intravenously injected murine CIMVs-MSCs 1 h after injection were localized in the vessel-rich organs such as lung and liver, small amount of CIMVs-MSCs reached spleen and brain (Fig. 8E). After 48 h CIMVs-MSCs were redistributed and localized in liver, lung, as well as in brain, heart, spleen and kidneys (Fig. 8F). These findings may be explained by an uptake of remaining in blood CIMVs-MSCs and their gradual renal excretion. Wiklander and colleagues conducted an extensive biodistribution investigation of EVs and showed that EVs were accumulated mainly in liver, spleen, gastrointestinal tract and lungs 24 h after the systemic injection^25^.

In our study we have showed that CIMVs-MSCs could be detected within the organ up to 14 days following administration via subcutaneous and intramuscular injection, most likely due to incorporation into the tissue and long half-life of the dye. Therefore, we suggest that the subcutaneous and intramuscular injection of CIMVs-MSCs is more suitable for the local therapy.

## Conclusions

CIMVs can be generated from human and murine MSCs. CIMVs retain the content, immunophenotype, and biological activity of the parental MSCs. CIMVs are similar in size to naturally produced microvesicles and do not contain the nuclei. One limitation to clinical exploitation of CIMVs derived from MSC was an absence of detailed characterization of the immune phenotypes. For this reason, we analyzed the immune modulatory properties of murine MSC and CIMVs-MSCs. The murine CIMVs-MSCs closely resemble parental MSCs and have similar phenotype and content of the biologically active molecules. CIMVs-MSCs also demonstrate suppression of antibody production similar to parental MSCs. There are evidences that the EVs-MSCs immunosuppressive effect is lower compared to parental MSCs. We observed that natural EVs derived from MSCs failed to suppress antibody production in vivo. Our study provides new evidence on the CIMVs as a perspective candidate of a cell-free therapeutics which retain the immunosuppressive activity of parental MSCs.

## Supplementary information

Supplementary information

## Data Availability

All data generated or analysed during this study are included in this published article (and its Supplementary Information files). The data that support the fndings of this study are available from the corresponding author upon request.
