# Supplementary material for: Immunosuppressive properties of cytochalasin B-induced membrane vesicles of mesenchymal stem cells: comparing with extracellular vesicles derived from mesenchymal stem cells
Source: Sci Rep. 2020 Jul 1;10:10740. doi: 10.1038/s41598-020-67563-9 (PMC7330035; doi:10.1038/s41598-020-67563-9)
Supplement: Supplementary file 1 — Supplementary information [file 41598_2020_67563_MOESM1_ESM.docx]

**Immunosuppressive properties of cytochalasin B-induced membrane vesicles of mesenchymal stem cells: comparing with extracellular vesicles derived from mesenchymal stem cells**

M.O. Gomzikova^1,2^, A.M. Aimaletdinov^1^, O.V. Bondar^1^, I.G. Starostina^1^, N.V. Gorshkova^1^, O.A. Neustroeva^1^, S.K. Kletukhina^1^, S.V. Kurbangaleeva^1^, V.V. Vorobev^1^, E.E. Garanina^1^, J.L.Persson^4^, J. Jeyapalan^5^, N.P.Mongan^5,6^, S.F. Khaiboullina^1,3^, A.A.Rizvanov^1,2^

**Supplementary Data**

**RESULTS**


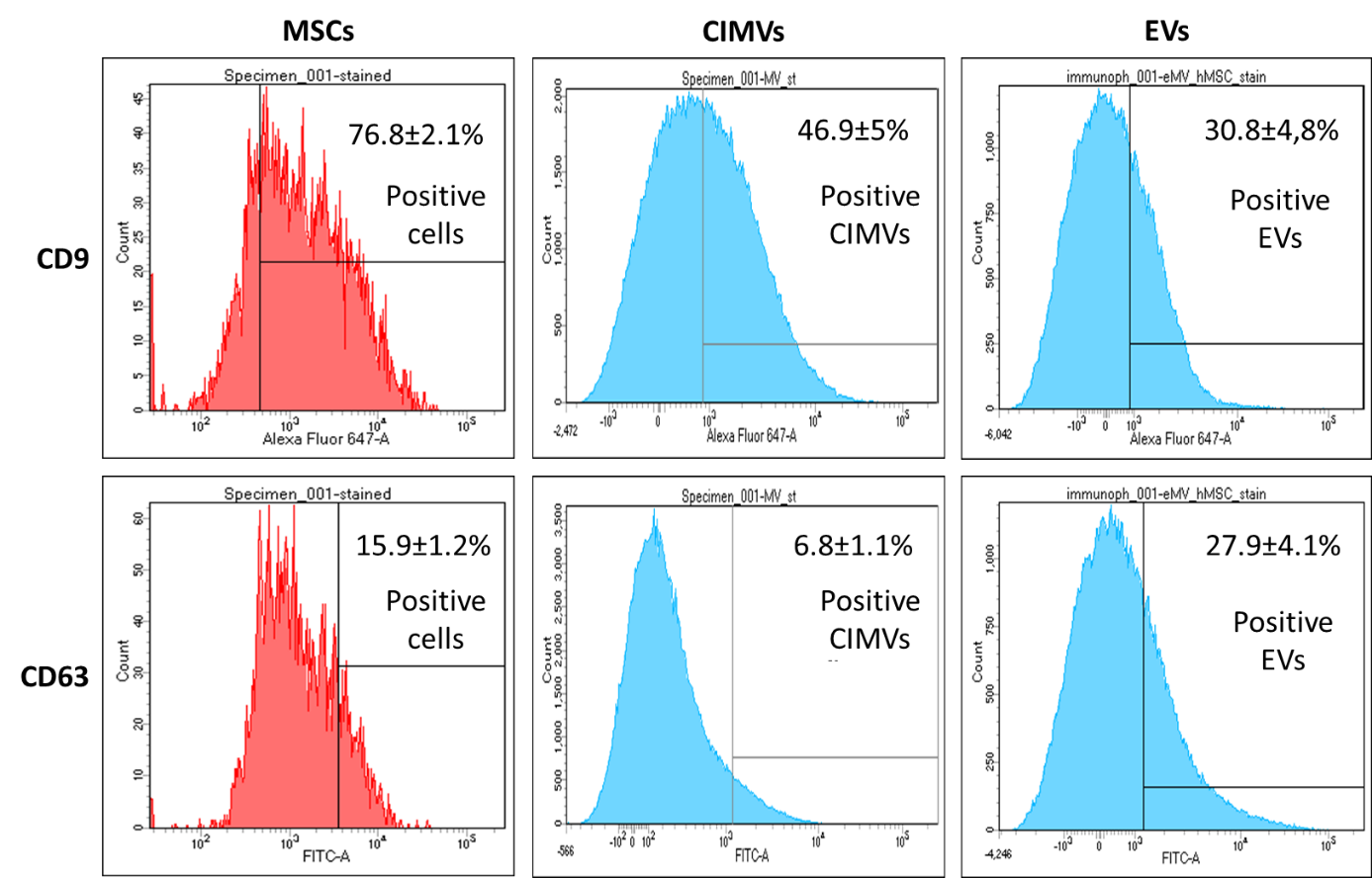


**Supplemental Figure 1**. Flow cytometry results of MSCs, CIMVs-MSCs and EVs immunostaining with monoclonal antibodies CD9 and CD63.

**Nucleic acid content of CIMVs**

CIMVs content was characterized using PCR. Total DNA was isolated and used as a template in PCR amplification of COI and 18S rRNA genes.

**
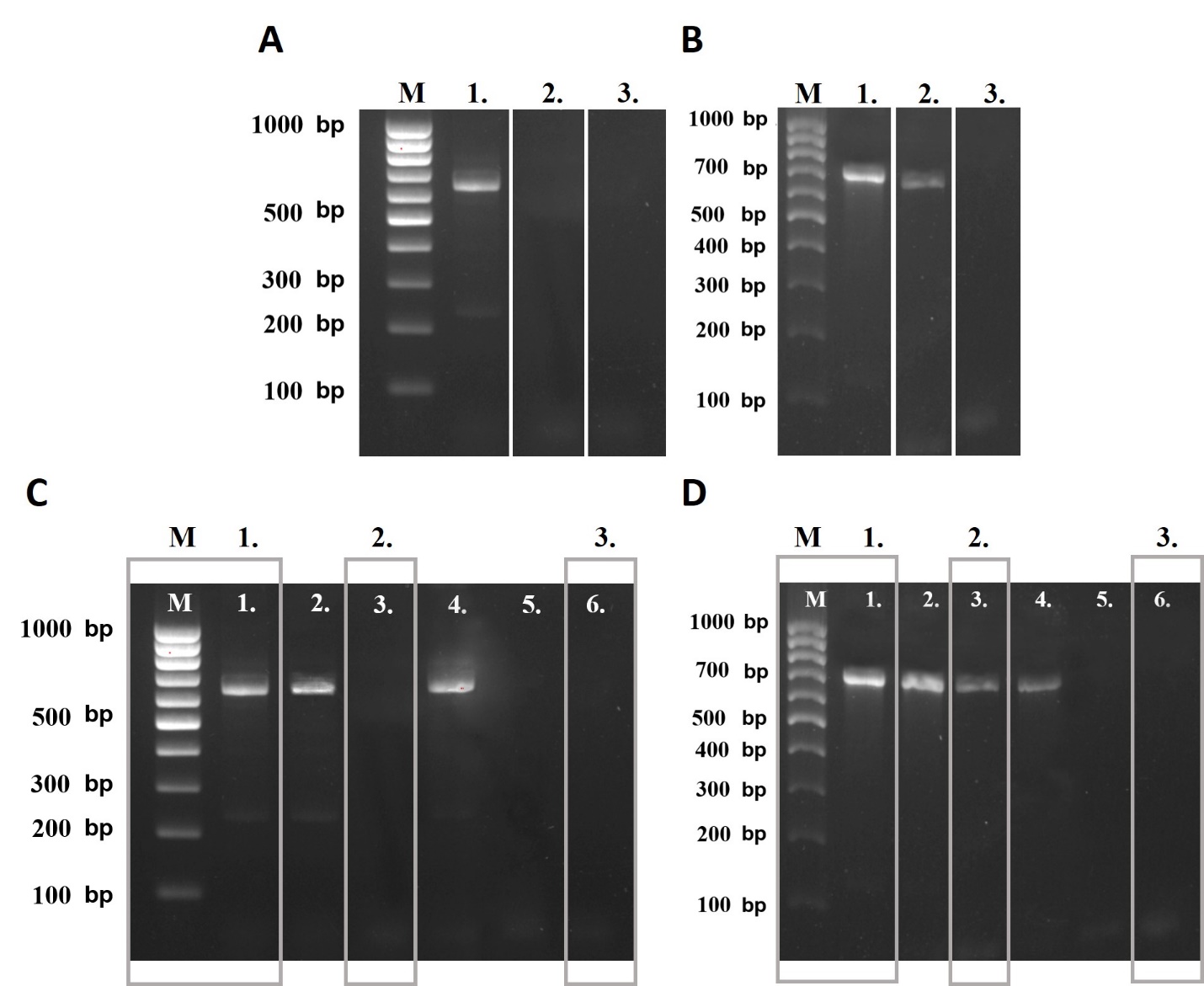
**

**Supplemental Figure 2**. Gel electrophoresis of PCR products using primers specific for 18S rRNA (A) and cytochrome oxidase I (COI) (B) genes. Lane M: molecular marker (100 base pair DNA ladder), Lane (1): PCR product of MSCs, Lane (2) PCR product of cytochalasin B induced membrane vesicles, Lane (3) negative control. The samples derived from the same gel. C,D - full-length gels are presented. Gray squares show the excised field of gels presented in A and B.

**Influence of the allogenic MSCs or CIMVs-MSCs on the neutrophil activity**

**
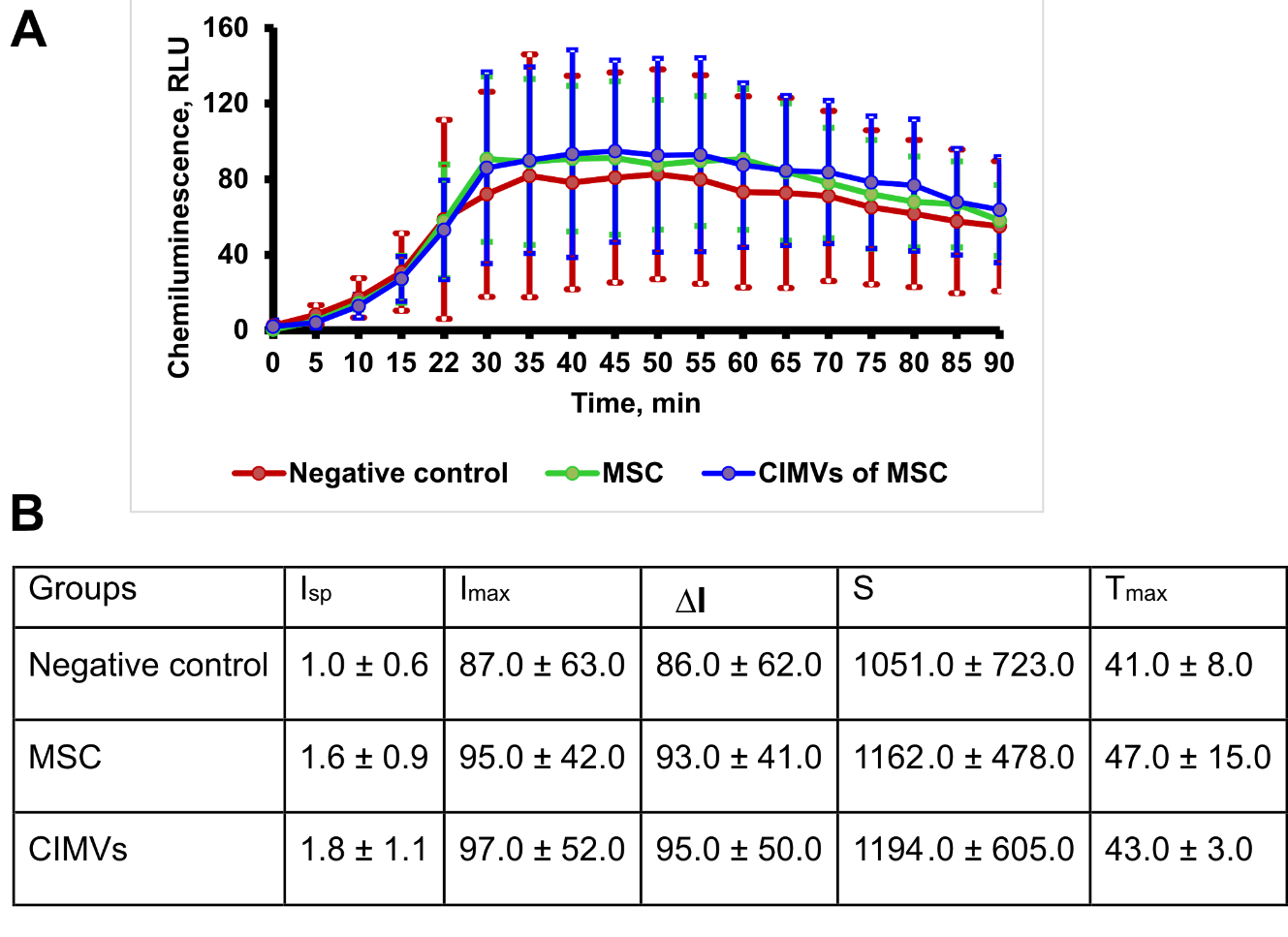
**

**Supplemental Figure 3.**Chemiluminescence analysis of neutrophils ROS release. (A) The dynamics of neutrophil chemiluminescence after stimulation with opsonized zymosan. Neutrophils were isolated from control (PBS injection) mice (red line) and pretreated with allogenic MSCs (green line), or CIMVs-MSCs (blue line). RLU – relative light units were measured by TecanNanoquant Infinite 200 Pro (Tecan, Switzerland). Data presented as Mean±SD. (B) The level of spontaneous chemiluminescence (I_sp_ - luminescence spontaneous), the maximum value of stimulated chemiluminescence (I_max -_ luminescence max), the intensity of stimulated chemiluminescence (∆I=I_max_ -I_sp_). ROS production was expressed as the total RLU accumulated in 90 min. T_max_ is the time that requires to reach the maximum intensity of chemiluminescence after the addition of the stimulus (opsonized zymosan).

**Effect of MSCs and CIMVs-MSCs on the macrophage activity**


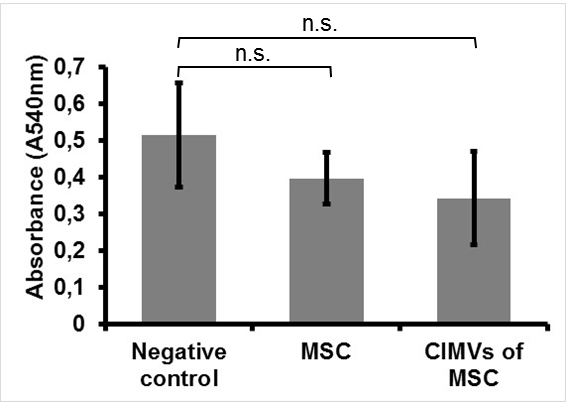


**Supplemental Figure 4.** The phagocytic activity of peritoneal macrophages from mice pretreated with allogenic MSCs, CIMVs-MSCs or PBS (negative control). Mice were injected with MSCs or CIMVs-MSCs i.v., and used for collection of macrophages from peritoneal lavage 24 hours later. Macrophages were incubated in the presence of 0.075% neutral red (Sigma, USA) for 4 hours and collected to determine PI. PI was analyzed by detection of optical density (540 nm) using Tecan Nanoquant Infinite 200 Pro (Tecan, Switzerland). PI was determined as a ratio of an optical density of the peritoneal macrophage lysate relatively to number of cells. n.s. - not significant.

**Effect of MSCs and CIMVs-MSCs on total leukocyte count in the spleen, thymus and bone marrow**


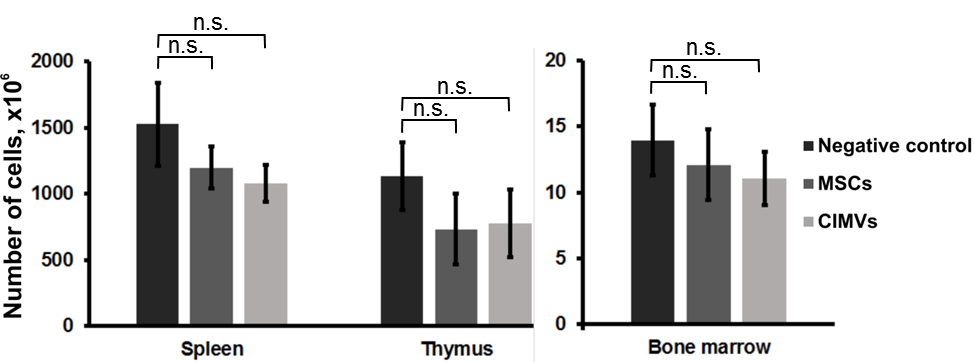


**Supplemental Figure 5.** The effect of MSCs and CIMVs-MSCs on total leukocyte count in spleen, thymus and bone marrow. Negative control – mice were injected i.v. PBS 24 hours before the analysis; MSCs - mice were injected i.v. MSCs (7.5x10^4^) 24 hours before the analysis; CIMVs-MSCs–mice were injected i.v. CIMVs-MSCs (15 µg) 24 hours before the analysis. Lymphoid organs (spleen, thymus and bone marrow) were collected and used to determine the weight before extraction of leukocyte. The leukocyte population was filtered (40 μm) and used to determine the cell count. n.s. - not significant.

**T-cell suppression assay *in vitro***


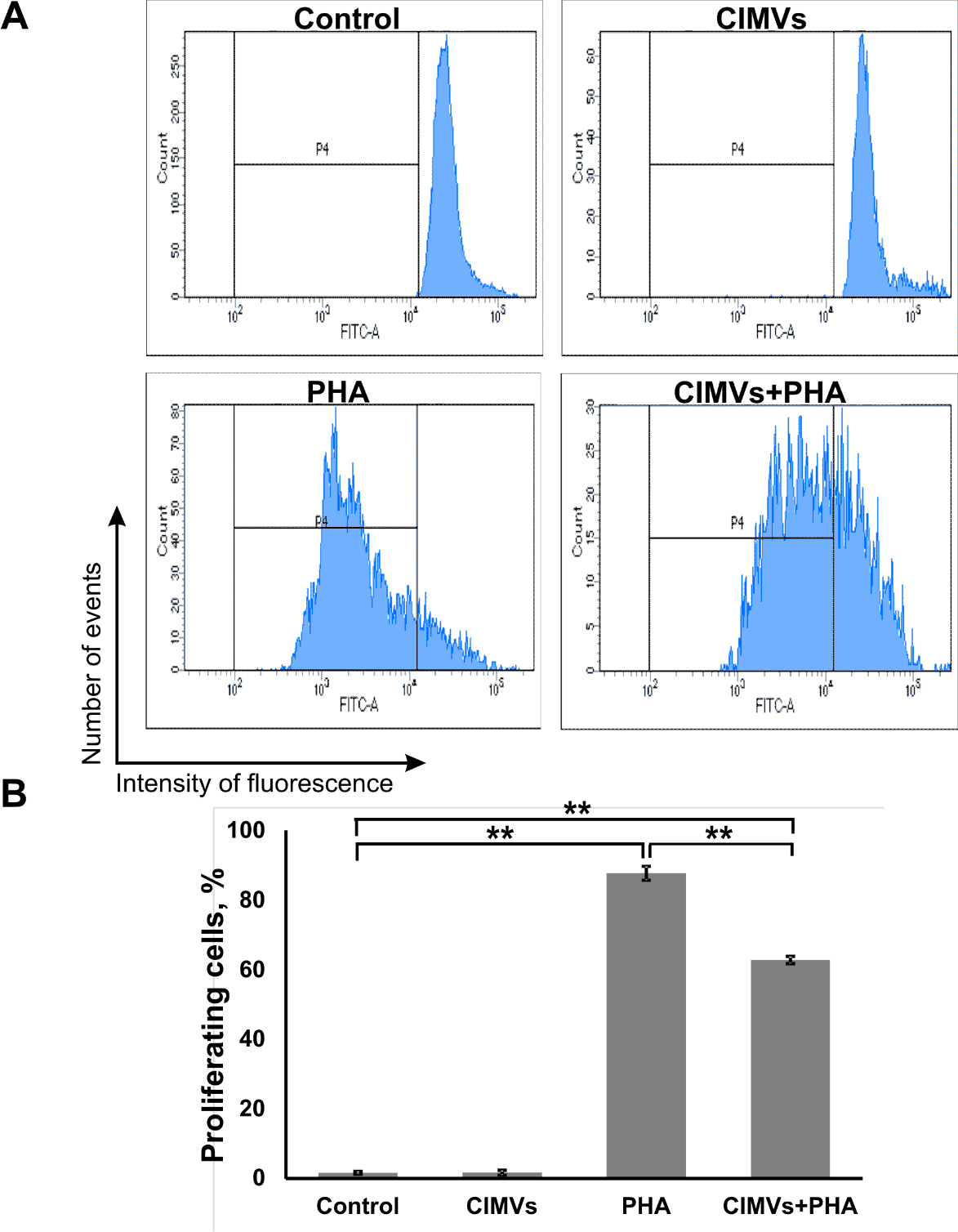


**Supplemental Figure 6.** The effect of CIMVs-MSCs on PHA-activated proliferation of T-cells. PBMCs were prestained with CFDA SE. Flow cytometry data (A). Histograms were generated using FACSDiva7 software (BD Bioscience, USA). Percent of T-cells with decreased CFDA SE fluorescence was taken to determine the T-cells proliferation rate (B). The data represents mean ± SD. (**) - level of significance <0.01.
